# Improving wheat as a source of iron and zinc for global nutrition

**DOI:** 10.1111/nbu.12361

**Published:** 2019-01-14

**Authors:** J. Balk, J. M. Connorton, Y. Wan, A. Lovegrove, K. L. Moore, C. Uauy, P. A. Sharp, P. R. Shewry

**Affiliations:** ^1^ John Innes Centre Norwich Research Park Norwich UK; ^2^ School of Biological Sciences University of East Anglia Norwich UK; ^3^ Department of Plant Science Rothamsted Research Harpenden UK; ^4^ School of Materials University of Manchester Manchester UK; ^5^ Photon Science Institute University of Manchester Manchester UK; ^6^ Department of Nutritional Sciences Kings College London UK

**Keywords:** bioavailability, biofortification, iron, phytic acid, wheat, zinc

## Abstract

Wheat is the staple food crop in temperate countries and increasingly consumed in developing countries, displacing traditional foods. However, wheat products are typically low in bioavailable iron and zinc, contributing to deficiencies in these micronutrients in countries where wheat is consumed as a staple food. Two factors contribute to the low contents of bioavailable iron and zinc in wheat: the low concentrations of these minerals in white flour, which is most widely consumed, and the presence of phytates in mineral‐rich bran fractions. Although high zinc types of wheat have been developed by conventional plant breeding (biofortification), this approach has failed for iron. However, studies in wheat and other cereals have shown that transgenic (also known as genetically modified; GM) strategies can be used to increase the contents of iron and zinc in white flour, by converting the starchy endosperm tissue into a ‘sink’ for minerals. Although such strategies currently have low acceptability, greater understanding of the mechanisms which control the transport and deposition of iron and zinc in the developing grain should allow similar effects to be achieved by exploiting naturally induced genetic variation. When combined with conventional biofortification and innovative processing, this approach should provide increased mineral bioavailability in a range of wheat products, from white flour to wholemeal.

## Introduction

It is difficult to overemphasise the global importance of deficiencies of mineral micronutrients, principally of iron and zinc, in human diets. It has been estimated that globally 43% of children and 29% of women of reproductive age have anaemia, and about half of these cases result from iron deficiency (WHO 2015). Zinc deficiency is associated with stunted growth in children under the age of 5 years and reported to affect approximately 155 million children globally (WHO 2013). In the UK, the zinc intake of around a quarter of adolescents is below the lower reference nutrient intake (LRNI; intakes below which are inadequate for most individuals), and the iron intake of over half of adolescent girls and over a quarter of adult females is below the LRNI (Roberts *et al.* 2018). Cereals such as wheat, rice and maize provide 30–35% of energy intake in the UK (Bates *et al*. 2014) and up to 60% of the daily calories in developing countries (Ritchie & Roser 2018), but conventional processing of the grains removes most of the micronutrients.

Many countries have mandatory fortification for iron and selected vitamins in flours from wheat, maize and rice (www.ffinetwork.org). Fortification of cereal flour with zinc is also practised in several countries, but largely on a voluntary basis (Brown *et al*. 2010). All white wheat flour milled in the UK is fortified in accordance with the Flour and Bread Regulations 1998 (http://www.legislation.gov.uk/uksi/1998/141/contents/made) with inorganic forms of iron at levels of 16.5 mg/kg (equivalent to levels in high extraction rate wheat flour), which then enters the food chain through bread, pasta, noodles, cakes, biscuits and a range of other products. In other parts of the world where wheat is a staple crop, such as north India and Pakistan, fortification is difficult to implement because milling is carried out domestically at a small scale as well as in large‐scale industrial mills. Hence, more innovative strategies are required to ensure sustainable micronutrient levels in those regions that need it most.

The provision of adequate minerals from bread and other cereal products is determined by their total amount in the grain and by their bioavailability. Although the latter can be increased, to a limited extent, by post‐harvest processing such as micro‐milling and fermentation, modern breeding approaches may be the only way to achieve the profound increases in the amounts of bioavailable iron and zinc that are required to meet the metabolic demands of the global population, by altering the localisation and form of the minerals in the grain.

## Location of iron and zinc in wheat grains

**Figure 1:**
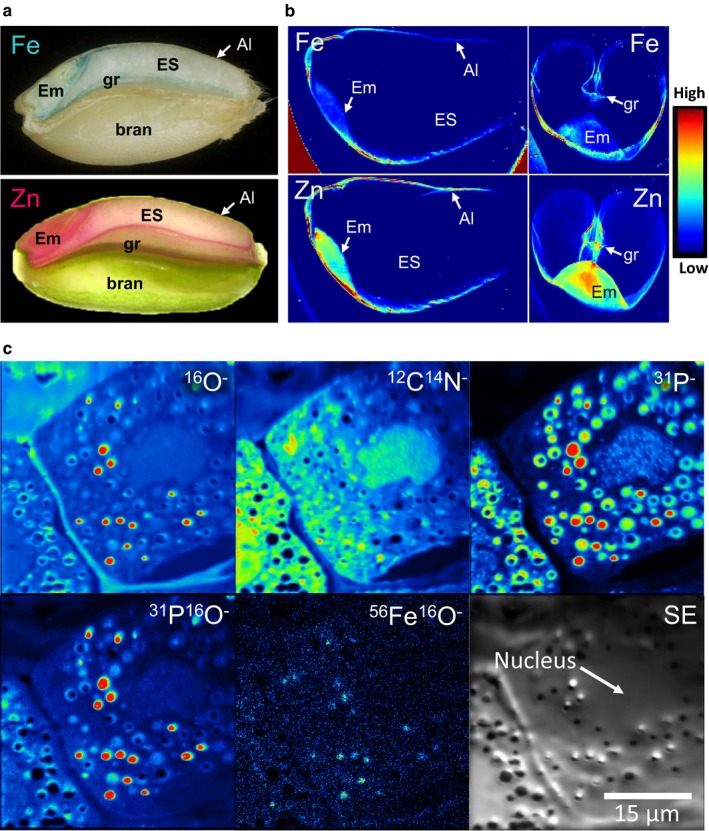
The locations of iron and zinc in wheat grain. (a) Transverse sections through wheat grains, showing the position of the embryo (Em), starchy endosperm (ES), aleurone (Al), groove (gr) and bran and the locations of iron (light blue from staining with Prussian blue in upper image) and zinc (red from staining with dithizone in lower image). (b) Heat map representation of the distribution of iron (Fe) and zinc (Zn) in longitudinal and transverse sections of wheat grain, revealed by X‐ray fluorescence. Taken from Neal *et al*. (2013) with permission. Labelling as in Panel (a). (c) NanoSIMS images of an aleurone cell of an immature wheat grain showing localisation of ^56^Fe^16^O^−^ to the phytin globoids (as indicated by the ^31^P^16^O^−^ image). White indicates high signal intensity. Taken from Moore *et al*. (2012) with permission. [Colour figure can be viewed at wileyonlinelibrary.com]

The wheat grain is a single‐seeded fruit, called a caryopsis. It contains a small embryo, which forms the new plant on germination, and a large storage tissue (the endosperm), which comprises mainly starch (a source of energy) and protein. These tissues are surrounded by protective layers derived from the seed coat (testa) and fruit coat (pericarp). Iron and zinc, together with other minerals, are concentrated in the embryo and in the outer layer of endosperm cells, called the aleurone. These distributions can be clearly seen in sections of a wheat grain using simple staining methods (Fig. 1a), but higher resolution is obtained by modern imaging systems such as Synchrotron X‐ray fluorescence (Fig. 1b) and NanoSIMS (secondary ion mass spectroscopy) (Fig. 1c) (Moore *et al*. 2012; Neal *et al*. 2013). The uneven distribution of the two minerals makes sense from a plant biology perspective: upon germination of the seed, the embryo will grow rapidly into a young seedling for which it requires enzymes that are dependent on iron, zinc and other cofactors (Bastow *et al*. 2018). The growth is sustained by energy and amino acids derived from the storage reserves (starch and protein) in the central starchy endosperm. However, because the starchy endosperm cells die during the later stages of grain maturation (Young & Gallie 2000), the starch and protein are mobilised as sugars and amino acids after lytic digestion by enzymes secreted from the aleurone layer and embryo. Interestingly, the two minerals differ slightly in distribution between the two tissues, with iron being more concentrated in the aleurone and zinc in the embryo (as shown in Fig. 1b). The biological significance of these differences in the locations of the two minerals is not known.

Traditional milling of wheat by grinding between stones produces wholemeal flour in which all parts of the grain are mixed and can be only partially separated by sieving. However, the introduction of roller milling at the end of the 19^th^ century enabled the precise separation of the starchy endosperm from the embryo (germ) and the outer layers (including the aleurone), which are usually recovered together as the ‘bran’. This resulted in the availability of affordable white bread, previously an expensive luxury, for the whole population. However, the removal of both the germ and the aleurone layer means that white flour has substantially lower contents of iron and zinc than wholemeal – for example, means of 6.7 mg/kg iron and 8.4 mg/kg zinc in white flour, compared to 28.2 mg/kg iron and 28.6 mg/kg zinc in the wholegrain (Tang *et al*. 2008). The contents of iron and zinc can partially be restored in white flour by enriching it with wheat germ as in ‘patent breads’ (Burnett 2005). In addition, numerous public health campaigns have been aimed at increasing the consumption of wholemeal products. Nevertheless, of the total bread‐making flour produced in the UK, less than 10% is wholemeal (NABIM 2017).

Roller milling also results in the removal of most of the anti‐nutrient phytic acid, which limits mineral bioavailability (as discussed below). Phytic acid (inositol hexakisphosphate) is the storage form of phosphorus of plants, which is another important element for seedling germination and growth.

## Naturally occurring iron and zinc compounds and their bioavailability

Iron and zinc are essential cofactors in a wide range of metabolic enzymes. Thus, these elements occur in protein‐bound forms in plants and animals. Iron can bind directly to the protein, or as an iron–sulphur cofactor or as haem (Balk & Schaedler 2014). The bioavailability of haem iron is high because it is very stable and is thought to be taken up by a specific transporter in the smaller intestine (Knutson 2017). However, plants contain very little haem iron (<0.1% w/v; Espinas *et al*. 2012) in contrast to animal tissues (notably meat and liver). Zinc binds to proteins directly, for example, in zinc‐finger structures.

Iron and zinc are transported in plants as soluble forms chelated by small organic molecules and reach the developing grain through specialised vascular tissues (xylem and phloem), which extend along the groove of the grain (Fig. 1a). The organic acids citrate and malate facilitate the transport of iron in the xylem while nicotianamine facilitates iron and zinc transport in the phloem and intracellularly (Connorton *et al*. 2017a). Iron–nicotianamine complexes are present in extracts of white wheat flour (Eagling *et al*. 2014a) and nicotianamine enhances the bioavailability of iron and zinc as demonstrated in mouse studies (Lee *et al*. 2009) and in Caco‐2 cells (Eagling *et al*. 2014b).

Plants have the capability to store iron either inside a shell formed by the protein ferritin or in bodies derived from vacuoles (Connorton *et al*. 2017a). Wheat grains are low in ferritin, but high‐resolution imaging techniques such as NanoSIMS show that iron is localised in small intracellular bodies (Fig. 1c). These bodies also contain phosphate in the form of phytic acid and are called phytin globoids. Phytic acid has a cyclic structure with six phosphate groups, each of which can bind a metal ion such as iron and zinc (Maga 1982). Neal *et al*. (2013) used extended X‐ray fluorescence fine structure spectroscopy (EXAFS) to show the presence of iron and zinc complexes in wheat aleurone cells. A further application of X‐ray fluorescence, X‐ray absorption near‐edge structure (XANES) imaging, gives information on the atomic ligands of the metals, confirming that most of the iron is bound to phytic acid in the aleurone (De Brier *et al*. 2016). This colocation poses a challenge for human nutrition because mineral–phytate complexes tend to be insoluble with low bioavailability in humans.

## Agronomic and selective breeding strategies to increase bioavailable forms of iron and zinc

In addition to improvements in yield, disease resistance and processing quality, there has been growing research interest over the past 20 years in improving the health benefits of cereal crops, including increasing their mineral and vitamin contents (Vasconcelos *et al*. 2017), an approach known as biofortification. However, this has focused on increasing the mineral content in the wholegrain, rather than on the starchy endosperm tissue (which may limit the bioavailability).

There are two main biofortification approaches: agronomy and genetics (including conventional breeding and genetic modification; GM). Using agronomic methods, the zinc content of grain can be increased by simply fertilising the plants with zinc salts; for example, foliar application of ZnSO_4_ increased total grain zinc by about 60% (Zhang *et al*. 2012). However, such agronomic practices are less effective for iron, except if combined with increased nitrogen fertilisation (Aciksoz *et al*. 2011) which may not be economically or environmentally acceptable. Conventional breeding has been used by workers at the International Crops Research Institute for the Semi‐Arid Tropics (ICRISAT, India) to develop varieties of sorghum and pearl millet with increased contents of iron and zinc, and at the International Maize and Wheat Improvement Center (CIMMYT, Mexico) to increase the zinc content of wheat grain (Velu *et al*. 2018).

The zinc biofortified lines from CIMMYT are currently being grown in Pakistan and India and have 20–40% higher zinc concentration and at least comparable grain yield to the best local cultivars (Velu *et al*. 2018). Furthermore, human intervention trials to determine the bioavailability of the zinc in the biofortified lines are currently being carried out in Pakistan (Lowe *et al*. 2018). However, despite a number of research programmes globally, including at CIMMYT, no high iron wheat lines have yet been developed by conventional breeding.

## Transgenic strategies to increase bioavailable forms of iron and zinc

A step change in our ability to biofortify crops has come from a much better understanding of how plants take up and distribute micronutrients, mainly through the identification of genes for mineral transport and the biosynthesis of organic metal chelators. This knowledge has been exploited in modern biotechnology approaches, demonstrating that it is possible to increase iron and zinc levels, not only in the wholegrain but also specifically in the starchy endosperm. In fact, this shows that there is no biological reason why iron and zinc cannot be concentrated in the starchy endosperm and hence white flour.

The proof‐of‐concept of transgenic approaches was initially demonstrated in rice. Increased expression of *NAS3*, one of three genes encoding nicotianamine synthase (NAS), led to a 2.2‐fold increase in the concentration of zinc and a 2.9‐fold increase in the concentration of iron in the grain (Lee *et al*. 2009). Furthermore, feeding anaemic mice this enriched rice resulted in greater increases in haemoglobin and haematocrit (the volume of red blood cells in blood) compared to when conventional rice was fed. This high bioavailability results from the fact that the starchy endosperm cells do not store phytate. The initial transgenic work on NAS in rice led to similar studies in other cereals including wheat (*e.g*. Masuda *et al*. 2009; Zheng *et al*. 2010; Johnson *et al*. 2011; Singh *et al*. 2017). The advantage of increasing nicotianamine levels is that it leads to increases in both iron and zinc because it serves as a chelator for both metals in their ionic forms.

**Figure 2:**
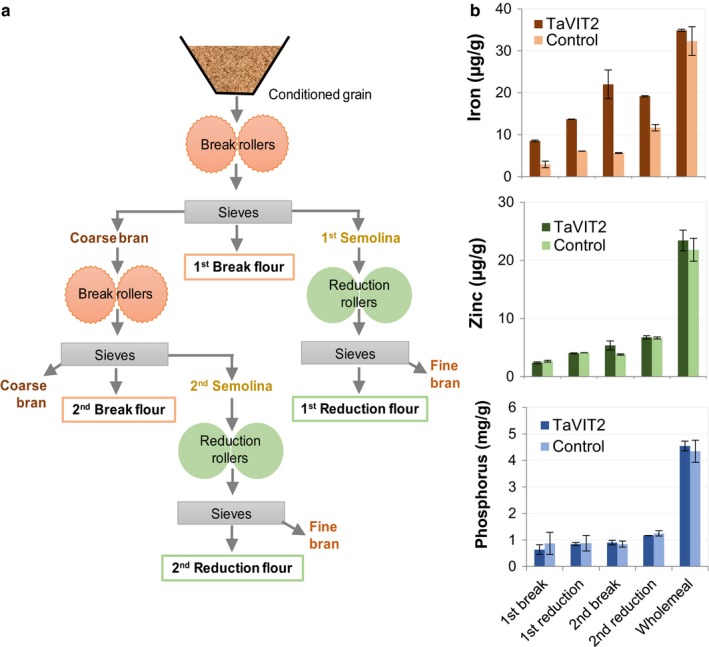
The contents of iron, zinc and phosphorus in white flour and wholemeal fractions from a transgenic wheat line expressing a vacuolar iron transporter (TaVIT2) and control wheat grain. (a) Milling scheme used to prepare white flour fractions (breaks 1 and 2, reductions 1 and 2) from the transgenic line TaVIT2 and control wheat grain using a Chopin CD1 laboratory mill. This scheme is a simplified version of the industrial roller milling process, with the first break and reduction flours being purer than the second break and reduction flours. (b) The contents of iron, zinc and phosphorus in white flour fractions (prepared using the scheme in part a) and wholemeal flour. Mineral contents were determined by Inductively Coupled Plasma – Optical Emission Spectrometry (ICP‐OES) analysis. Bars indicate mean ± standard error of the mean (SEM) of two technical replicates. [Colour figure can be viewed at wileyonlinelibrary.com]

By contrast, redirecting minerals into the starchy endosperm cells by overexpressing metal transporter genes leads to increases in single minerals, due to the high specificity of metal transporters, unless several genes are overexpressed together. For example, expression of the barley Metal Tolerance Protein 1 (*HvMTP1*), under the control of a starchy endosperm‐specific promoter, significantly increased the zinc content in the endosperm of barley grains (Menguer *et al*. 2018), while expression of a wheat Vacuolar Iron Transporter (*TaVIT2*) using a similar promoter more than doubled the iron content of the white flour fraction (Connorton *et al*. 2017b). This is illustrated in Fig. 2, which compares the contents of iron, zinc and phosphorus in bran and flour fractions of a *TaVIT2* transgenic line with control wheat grain milled on a laboratory roller mill. It is notable that the increased content of iron in the white flour (break and reduction) fractions in the transgenic line is not accompanied by an increase in phosphorus, showing that iron can accumulate in the endosperm without being associated with phytic acid.

More recently, Wu *et al*. (2018) have shown that preventing iron storage in the vacuoles, while at the same time overexpressing the iron storage protein ferritin specifically in the starchy endosperm cells, greatly increased iron in polished rice.

Hence, it is now accepted that transgenesis can be used to increase the contents of bioavailable iron and zinc in the starchy endosperm of cereals (white flour of wheat and polished white rice) by several‐fold, by redirecting mineral transport and/or providing a sink to sequester the iron.

## Post‐harvest improvements of iron and zinc bioavailability

It is probable that modern breeding approaches discussed above will be combined with novel processing approaches to increase the content of bioavailable minerals in wheat‐based foods, ranging from wholemeal to white flour products. The two most promising of these are mechanical treatments and fermentation. Mechanical treatments, particularly micro‐grinding, have been discussed in a recent article in this journal (Aslam *et al*. 2018). Plant cell walls are resistant to digestion in the gastro‐intestinal tract and therefore mineral bioaccessibility from wheat aleurone cells may be limited (Latunde‐Dada *et al*. 2014; Edwards *et al*. 2015). Enzymatic treatment and micro‐milling techniques designed to disrupt the aleurone cell walls enhance the release of iron from wheat flour during *in vitro* digestion and increase iron uptake by intestinal epithelial cells (Latunde‐Dada *et al*. 2014). This suggests that disruption of the aleurone cell walls may be an effective approach to increase iron bioavailability from wheat products.

Many microorganisms secrete phytase enzymes, which can release minerals from phytate complexes, particularly microorganisms present in sourdough systems (Katina *et al*. 2005; Rodriguez‐Ramiro *et al*. 2017). Hence sourdough wholegrain products may have increased mineral bioavailability. However, whereas this approach may increase mineral bioavailability in foods made from wholegrain and high extraction flours, it is not relevant to white flour products which are dominant in most countries.

## Future perspectives

Although transgenic strategies provide exciting opportunities to make dramatic increases in the contents and bioavailabilities of minerals in white flour products, it must be borne in mind that transgenic crops have limited acceptability by consumers and regulatory bodies, particularly in the European Union but also in many less developed countries. Furthermore, the recent (July 2018) ruling from the European Court of Justice that gene‐edited crops, which do not contain foreign DNA, should be considered genetically modified organisms (GMOs) (https://bit.ly/2RZFzmB) suggests that restrictions on growth and marketing are unlikely to be relaxed in the near future.

Although conventional mutagenesis remains outside GM legislation, most mutations result in loss, or reduced, gene expression and the application of mutagenesis to biofortification is therefore a challenge. Nevertheless, this is likely to be the most promising route for developing biofortified wheat in the future. Elucidation of the pathways and mechanisms of iron and zinc transport and deposition in the developing grain should identify genes encoding key transporters, or other factors, that can be downregulated or switched off, to redirect minerals into the starchy endosperm. This will be facilitated by the availability of comprehensive libraries of wheat mutants (Krasileva *et al*. 2017). Combined with conventional biofortification and innovative processing this should provide increased mineral bioavailability in a range of wheat products, from white flour to wholemeal.

## Conflicts of interest

The authors have no conflicts of interest to disclose.
